# Prevalence, diversity, and parasitism of tailed prophages in *Vibrio harveyi*

**DOI:** 10.1128/msphere.00228-25

**Published:** 2025-08-25

**Authors:** Ruijie Ma, Huiying Zhong, Rui Zhang

**Affiliations:** 1 College of Civil and Transportation Engineering, Shenzhen University 605391 , Shenzhen, China; 2 Archaeal Biology Center, Synthetic Biology Research Center, Shenzhen Key Laboratory of Marine Microbiome Engineering, Key Laboratory of Marine Microbiome Engineering of Guangdong Higher Education Institutes, Institute for Advanced Study, Shenzhen University 620539, Shenzhen, China; University of Michigan, Ann Arbor, Michigan, USA

**Keywords:** *Vibrio*, prophage, plasmid-phage, *Caudoviricetes*, CRISPR-Cas systems

## Abstract

*Vibrio harveyi*, a pathogenic bacterium, contains prophages that significantly influence its pathogenesis and evolutionary traits. Investigating the prevalence, evolution, and ecological roles of these prophages is of great importance as *V. harveyi* is responsible for luminous bacteriosis in aquatic organisms. In this study, 13 tailed prophages were identified from 55 globally sourced *V. harveyi* genomes, with prophage–bacterium junctions precisely annotated. These prophages exhibited distinct parasitic mechanisms, including Mu-type transposition, site-specific recombination, and a plasmid-like non-integrated state, reflecting their adaptive plasticity. Proteome-based phylogenetic analysis classified these prophages into eight subfamilies and nine genera, with half representing novel taxonomic singletons. Network analysis of *V. harveyi* prophages and a large set of prophages across *Vibrio* species revealed distinct prophage distribution patterns, including broad cross-species dissemination and clade-specific or strain-specific colonization. Further genomic analysis identified homologs of experimentally validated virulence factors associated with motility and biofilm formation, suggesting a potential role of these prophages in enhancing bacterial pathogenicity and adaptive fitness. CRISPR spacer matching provided the intra-species lytic history for 7 out of 13 identified prophages, underscoring their involvement in horizontal transfer of virulence traits. In summary, this study established a comprehensive genomic database of *V. harveyi* prophages, shedding light on their diversity, prevalence, and parasitic strategies.

**IMPORTANCE:**

Understanding how prophages parasitize *Vibrio harveyi* holds significant commercial implications, given the pathogen’s notoriety for inducing vibriosis across diverse aquatic species and causing substantial economic losses in the global aquaculture industry. We report here 13 well-curated prophage genomes identified from 55 globally collected *V. harveyi* genomes. Notably, these prophages exhibited previously unrecognized genomic diversity, along with distinct parasitic strategies and hierarchical distribution patterns. In-depth analysis of their genetic profiles identified multiple homologs of experimentally validated virulence determinants involved in regulating bacterial motility and biofilm formation. Lytic history was detected for over half of these prophages, suggesting their role in driving the dissemination of virulence traits within the species.

## INTRODUCTION

*Vibrio*, a group of gram-negative bacteria found in aquatic and marine environments, generally constitutes a minor component (up to 2%) of natural microbial communities, but can occasionally exceed 50% during blooms (1). This rapid proliferative capacity is attributed to *Vibrio*’s metabolic versatility and short doubling time under favorable conditions *in situ* (2, 3), traits that also facilitate its isolation and cultivation in the laboratory. Consequently, *Vibrio* has emerged as one of the most extensively studied bacterial model organisms over the past century (4). Significantly, certain *Vibrio* species act as opportunistic pathogens that may have adverse effects on marine animal hosts (5). *Vibrio harveyi* has been documented to cause severe pathological effects in economically important species, including prawns, spiny lobsters, and pearl oysters (6–8). The disease caused by *V. harveyi*, termed luminous bacteriosis or simply vibriosis, has led to high mortality rates in marine invertebrates and substantial financial losses to global aquaculture industries.

The primary mode of virulence of *Vibrio* species involves the secretion of extracellular enzymes and effector molecules, such as gelatinase, caseinase, phospholipase, hemolysin, chitinase, and siderophores (9). Bacterial virulence factors (VFs) are often acquired through horizontal gene transfer (HGT) via mobile genetic elements, such as plasmids and prophages (temperate phage genomes within bacterial genomes) (10). Temperate phages adopt dual lifecycles: lytic replication (immediate phage proliferation with host cell lysis) or lysogenic latency (phage genome integration into the host chromosome or maintenance as an episomal plasmid). During lysogeny, prophage-encoded repressor proteins prevent activation of lysis-related genes until environmental stressors (e.g., heat, pH changes, UV radiation) trigger the switch to the lytic cycle (11).

Previous studies implicated the role of temperate phage VHML (*Vibrio harveyi* myophage-like) in *V. harveyi* pathogenesis. Through lysogenic conversion, VHML transforms avirulent strains into virulent phenotypes, causing lethal effects on penaeid larvae in comparison with the isogenic strains without the presence of VHML (12). Subsequent research confirmed that VHML infection upregulates hemolysin production in the host bacteria (13). These findings underscore the need to investigate prophage prevalence in *V. harveyi* and identify putative prophage-encoded virulence determinants. To this end, this study analyzed 55 *V*. *harveyi* genomes from the National Center for Biotechnology Information (NCBI) RefSeq database and precisely annotated the genomic boundaries (i.e., prophage–bacterium junctions) of all *Caudoviricetes* prophages within these genomes. By leveraging this rigorously curated prophage data set with bioinformatic approaches, our study provided insights into the genomic diversity, prevalence patterns, and parasitic strategies of these tailed prophages.

## RESULTS AND DISCUSSION

### Overview of *V. harveyi* prophages

**Fig 1:**
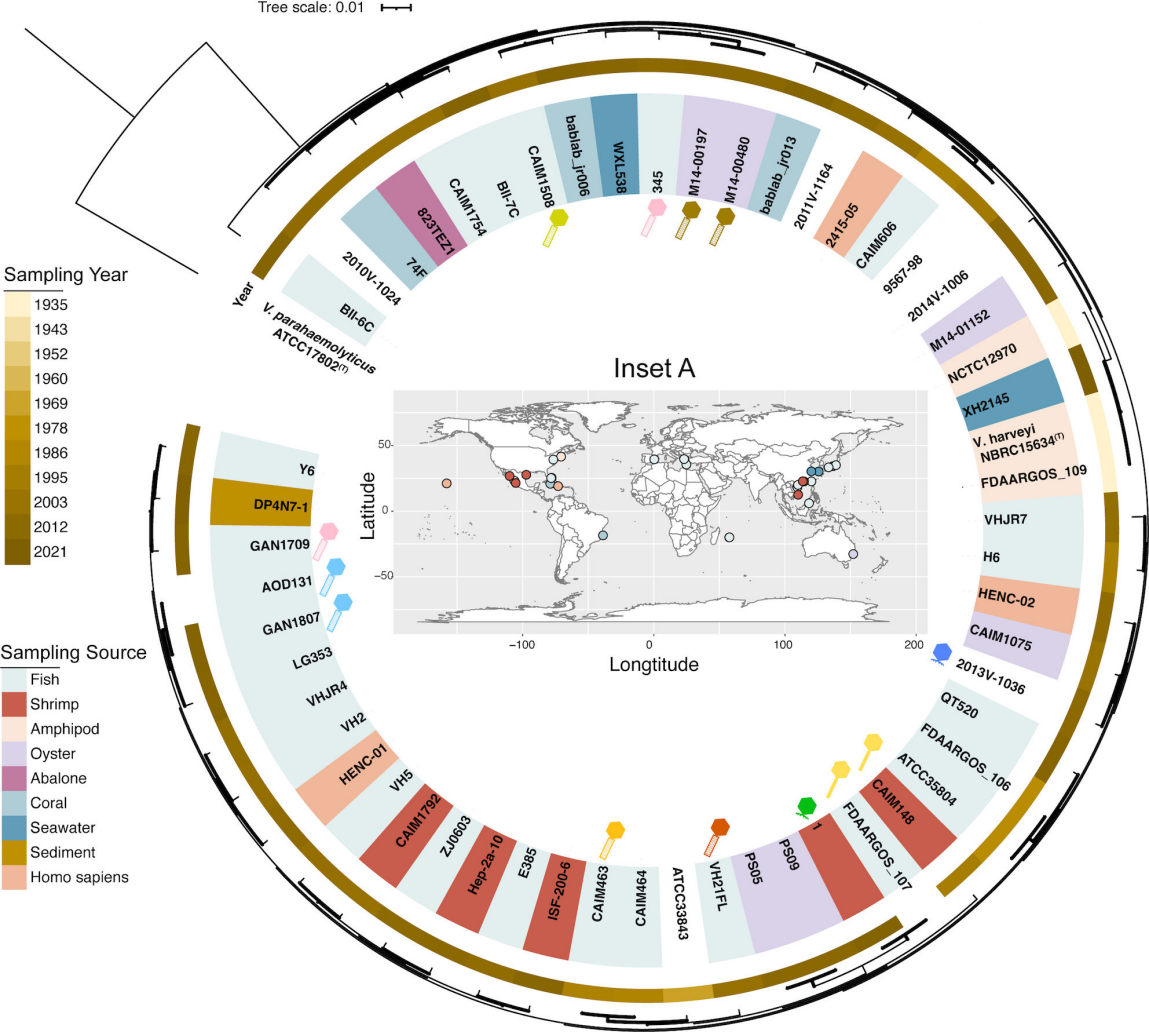
The whole-genome phylogeny of 55 *V*. *harveyi* genomes, with *Vibrio parahaemolyticus* ATCC 17802^T^ as the outgroup. Metadata of bacterial isolation sources and sampling years were displayed alongside the phylogeny. Tailed prophages identified in *V. harveyi* genomes were illustrated as schematic representations, with color coding corresponding to phage genus-level classification. Inset A: Geographic distribution of *V. harveyi* genomes and their isolation sources, created using ggplot2 and mapdata packages in R.

*V. harveyi* genomes available in the NCBI RefSeq database (accessed 1 March 2023, *n* = 59) were retrieved. To confirm their taxonomic classification, we performed genome-based DNA-DNA hybridization (DDH) analysis (14). According to the established bacterial species boundary threshold of ≥70% pairwise DDH similarity (15), we confirmed that 55 *V*. *harveyi*-like genomes shared 89.5% ± 3.1% (mean ± standard deviation [s.d.]) DDH similarity with the type strain NBRC 15634 (RefSeq: GCF_000400305.1). These *V. harveyi* genomes, collected worldwide over 86 years (1935 to 2021), were predominantly isolated from diseased or moribund fish, shrimp, and oysters (Fig. 1; Table S1).

**Fig 2:**
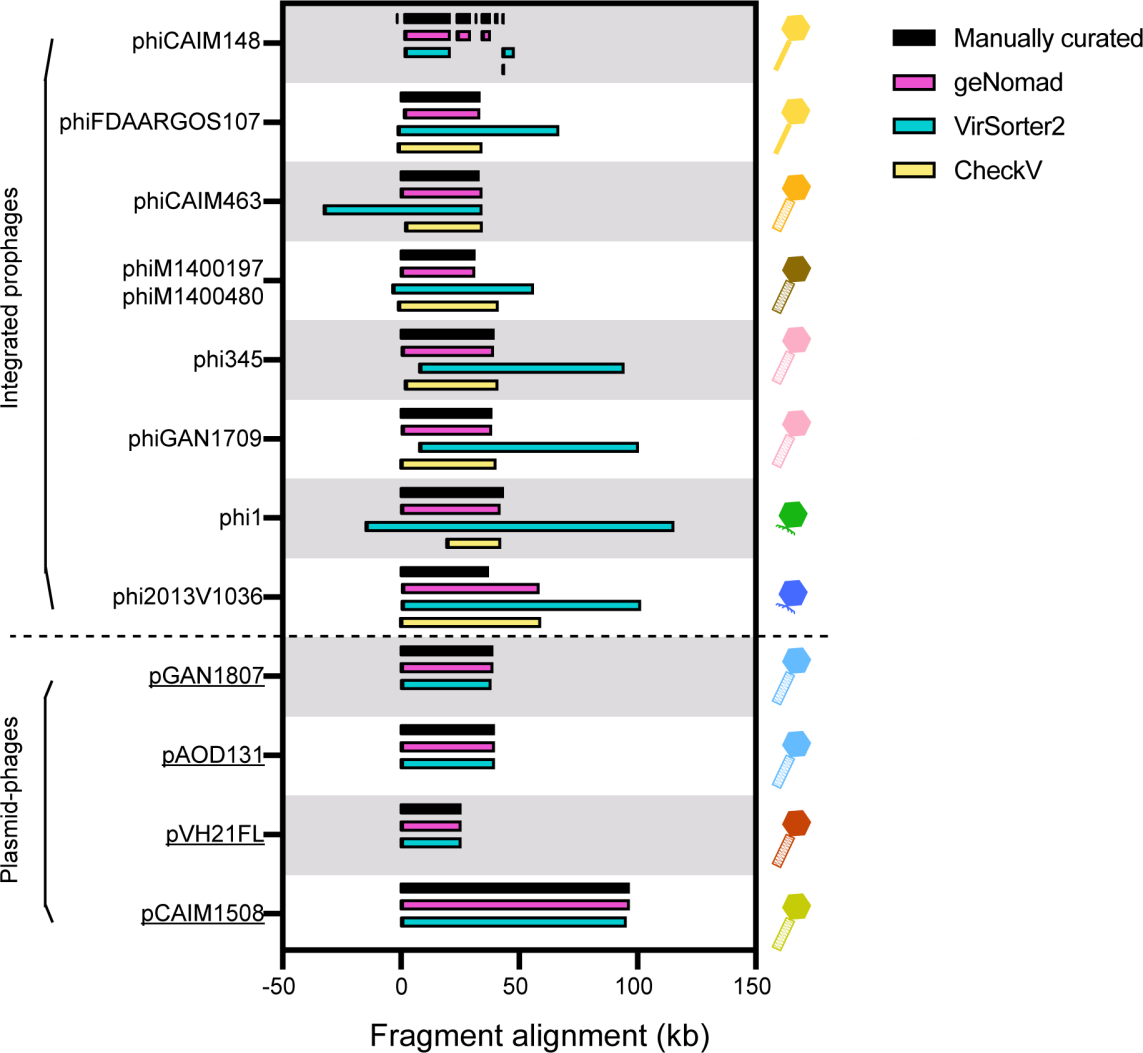
Prophage border annotation by manual inspection and bioinformatics tools. Fragment alignment between manually inspected prophage regions (black bars) and computational predictions from geNomad (magenta), VirSorter2 (cyan), and CheckV (yellow). Prophage nomenclature follows: “phi” for integrated prophages and “p” for plasmid-phages, followed by host strain identifiers.

For prophage identification, we combined manual inspection (identifying clustered phage-related genes in bacterial annotations) with computational predictions from three phage detection tools (CheckV, Virsorter2, and geNomad [16–18]) (Fig. 2). Genomic boundaries for each prophage candidate were manually verified by comparing the parental bacterial genome with an isogenic prophage-free reference genome, with precise coordinates of each prophage documented in Table S2. Our analysis identified 13 tailed prophages: nine integrated prophages flanked by bacterial sequences and four extrachromosomal phage DNA molecules (plasmid-phages) without adjacent host sequences. Integrated prophages averaged 36.8 ± 4.2 kb in length, while plasmid-phages showed a greater size range, varying from 25.7 kb (pVH21FL) to 96.8 kb (pCAIM1508).

### Parasitic strategies of *V. harveyi* prophages

**Fig 3:**
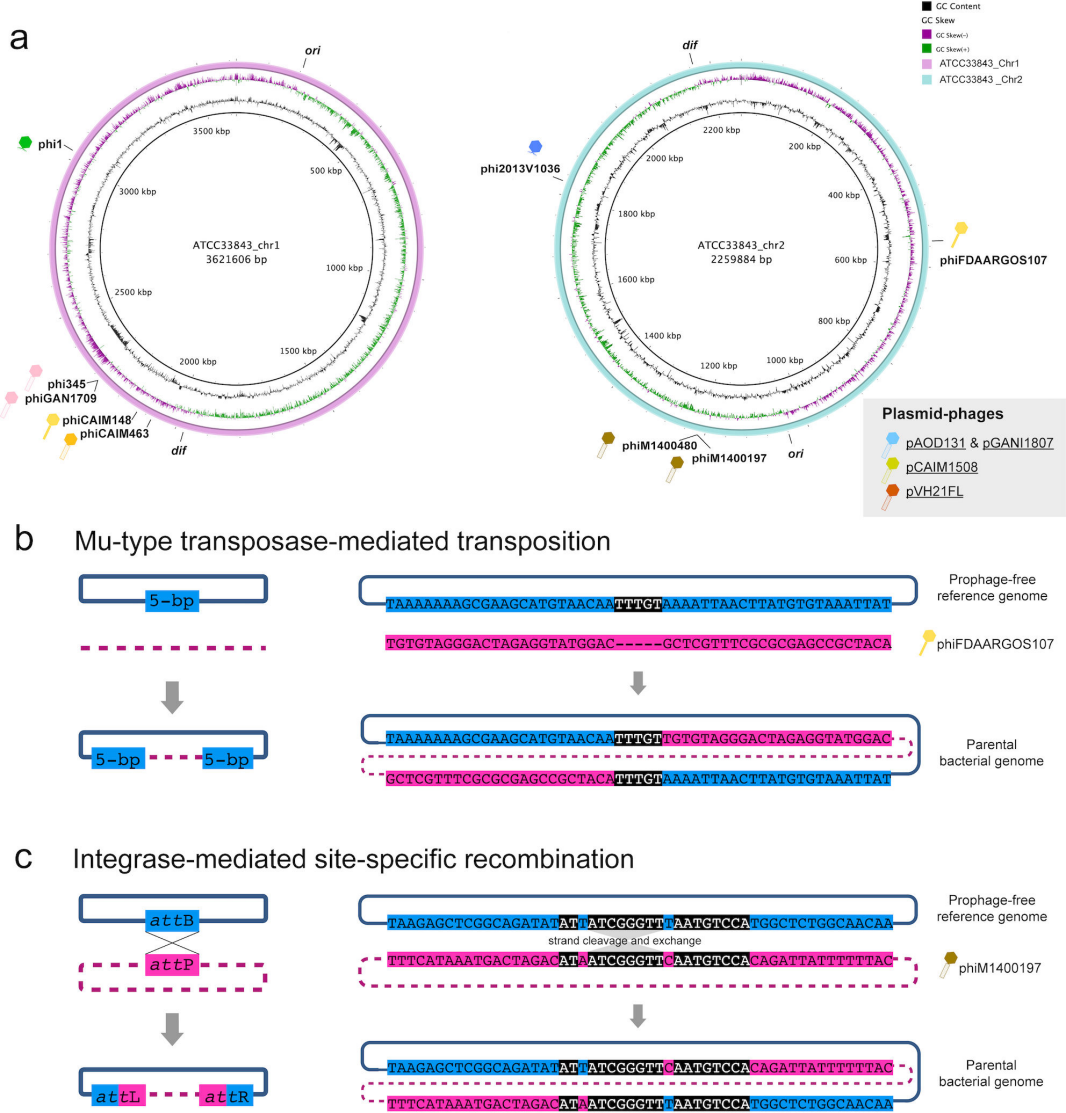
Prophage integration patterns. (**a**) Chromosomal integration sites mapped onto a fully sequenced reference genome, *V. harveyi* ATCC 33843 (RefSeq: GCF_000770115.1). Conserved chromosomal loci, *ori* (the origin of replication) and *dif* (deletion-induced filamentation), were marked as reference points. (**b**) Transposase-mediated transposition generating 5-bp target site duplications (black background). (**c**) Integrase-mediated site-specific recombination between phage (*att*P) and bacterial (*att*B) attachment sites, forming hybrid *att*L and *att*R junctions. Predicted *att* sites (black background) among the bacterial genome (blue background) and temperate phage genome (pink background) were shown.

All *Vibrio* spp. genomes contained two chromosomes: Chromosome I (Chr1) that primarily harbored essential genes, and Chromosome II (Chr2) that encoded a greater number of accessory genes (19). Integrated prophages were identified at seven distinct loci distributed across both *V. harveyi* Chr1 and Chr2 (Fig. 3a). The four plasmid-phages exhibited a unique parasitic strategy—they neither replicated their genomes through lytic development nor integrated into host chromosomes for synchronized replication during lysogeny. Instead, their DNA persisted as an extrachromosomal, plasmid-like state, representing a phenomenon termed pseudolysogeny (20). In this state, the phage genomes were maintained as non-integrated, non-replicating preprophages that could potentially enter either lytic or lysogenic cycles when environmental conditions or host nutritional status became favorable (21).

**Fig 4:**
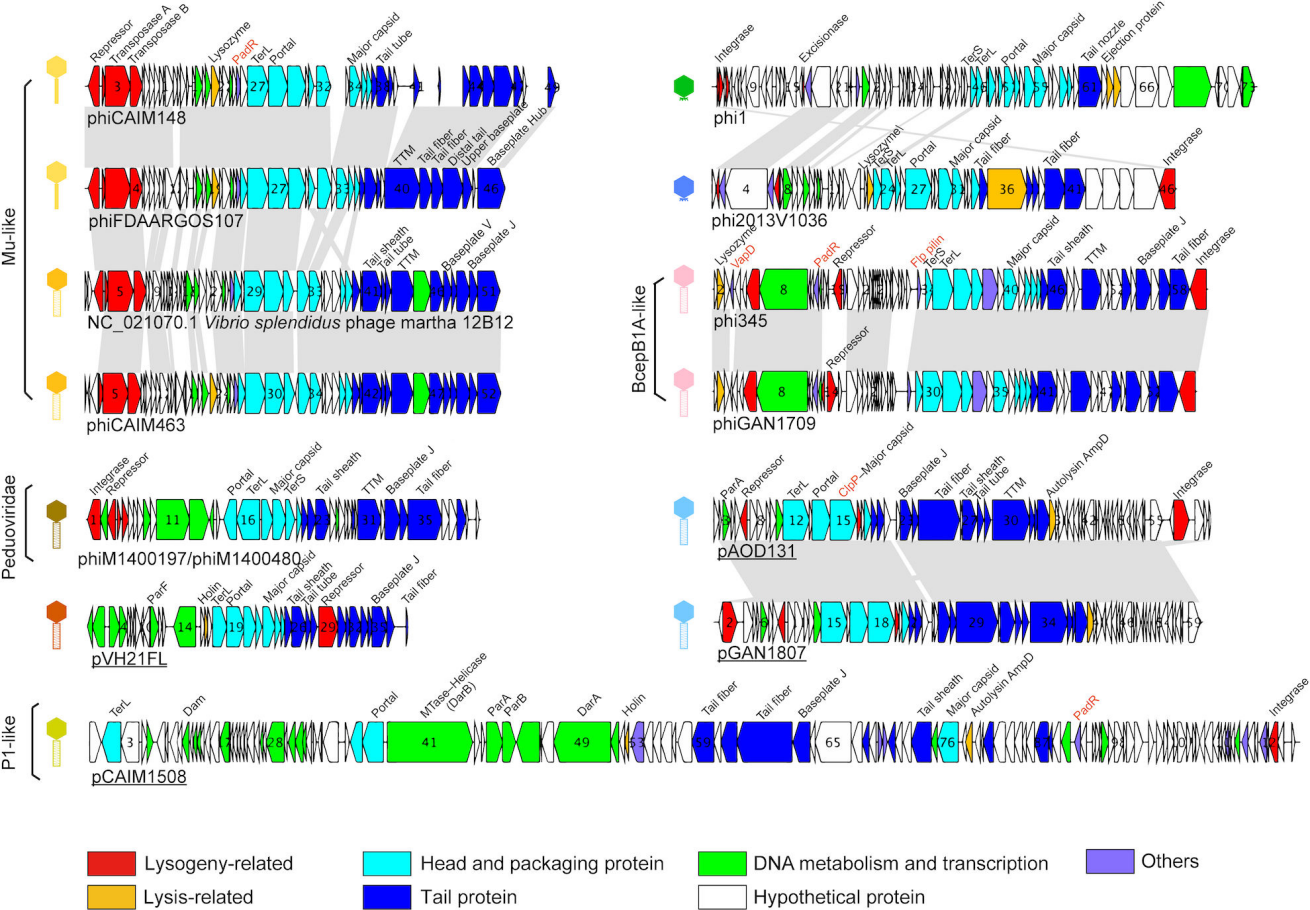
Genomic architecture of *V. harveyi* prophages. Integrated prophages were displayed in their native chromosomal context with host flanking sequences excluded, while plasmid-phages (underlined) existed as discrete contigs within bacterial genomes with no adjacent host sequences identified. Predicted morphotypes (siphophages, myophages, and podophages) were illustrated schematically. Genes functional categories were color-coded (red, lysogeny-related; orange, lysis-related; cyan, head and packaging; blue, tail; green, DNA metabolism and transcription; white, hypothetical; purple, others). Homologous genes were linked using gray shading (BLASTp E-value < 0.001 and bit-score ≥ 40). Gene products homologous to experimentally validated virulence factors were highlighted in orange. PadR, phenolic acid decarboxylase regulator; ClpP, caseinolytic protease proteolytic subunit; VapD, virulence-associated protein D; Flp pilin, fimbrial low-molecular-weight protein.

Among the identified prophages, phiCAIM148, phiCAIM463, and phiFDAARGOS107 were characterized as Mu-like due to their encoding of a pair of transposase genes. Since the prototypical phage Mu integrated its genome into the host chromosome via transposition (22), these prophages likely employed a similar mechanism. This hypothesis was further supported by the detection of identical 5-bp direct repeats flanking both ends of these prophage genomes (Fig. 3b), characteristic of Mu-type transposition through the “nick-join-repair” pathway (23). In contrast, non-Mu-like prophages (with the exception of plasmid-phage pVH21FL) encoded integrase (Int), which enabled Int-mediated site-specific recombination (Fig. 3c). Typically, these Int-encoding phages targeted a specific bacterial genomic fragment (*att*B) that precisely matched phage attachment site (*att*P). The recombination between *att*B and *att*P generated hybrid *att*L and *att*R sites flanking the integrated prophage. Since *att*P is located adjacent to the *int* gene (20), this recombination would position the *int* gene next to host flanking sequences. This characteristic genomic arrangement had been observed in all non-Mu-like integrated prophages but was absent in plasmid-phages (Fig. 4), confirming that the latter maintain their DNA in a non-integrated state.

Through comprehensive characterization of 5-bp direct repeats in Mu-like prophage genomes and *att*L/R sites in Int-encoding prophages, this study established a curated genomic collection of *V. harveyi* tailed prophages. Such a comprehensive data set provided accurate genomic references that underpinned bioinformatics analyses presented herein.

### Taxonomic diversity and novelty of *V. harveyi* prophages

**Fig 5:**
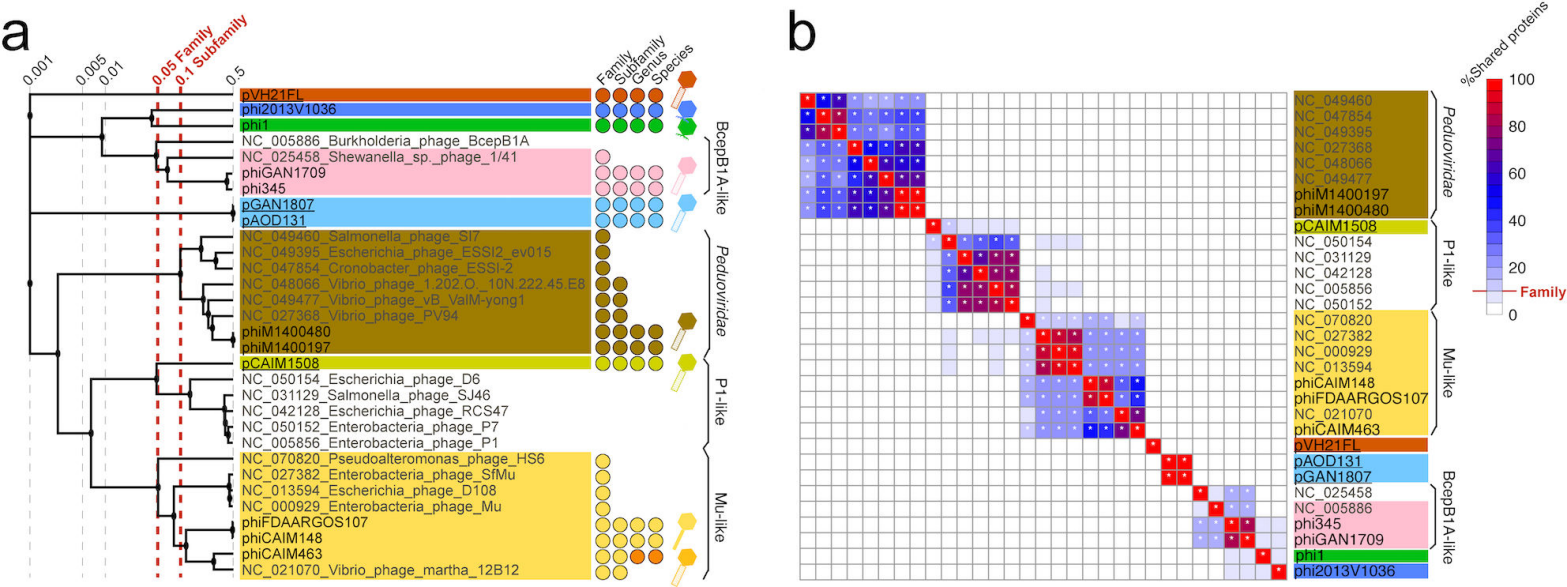
Taxonomic classification of *V. harveyi* prophages. (**a**) The ViPTree based on proteome-wide comparisons between *V. harveyi* prophages and reference phage genomes from RefSeq release 220. (**b**) Protein-sharing heatmap highlighting the proportion of orthologous gene content empirically used for phage family-level classification (≥10% shared proteins, marked with asterisks).

A viral proteome-based phylogenetic tree (ViPTree) was constructed to illustrate evolutionary relationships among these prophages (Fig. 5a). This approach overcame limitations of traditional marker gene-based phylogenetics, particularly when analyzing distantly related phages with low nucleotide-level identities (24, 25). Phage family-level and subfamily-level classifications were delineated using ViPTree branch length thresholds of 0.05 and 0.1 (26), respectively. Phages within the same family were empirically defined as sharing ≥10% orthologous genes, which further supported our classification, as evident by their protein-sharing profiles (Fig. 5b). Together, these criteria categorized the *V. harveyi* prophages into eight families and eight subfamilies. Subsequent genus/species demarcation with widely accepted average nucleotide identity thresholds (70% for genus and 95% for species) (25) identified nine genera and nine species.

*Vibrio* spp. represented extensively studied model marine bacteria with hundreds of phage isolates (vibriophages) reported to date. Strikingly, half of the identified prophage subfamilies appeared as novel singletons (including plasmid-phages pVH21FL, pGAN1807, pAOD131, and podophages phi1 and phi2013V1036), lacking homologous reference phage genomes from the RefSeq database (release 220). This discrepancy likely reflected isolation biases: researchers prioritize virulent phages over temperate phages due to the former’s therapeutic potential against pathogenic *Vibrio* species (19). The morphology of phage lysis zones (plaques) provided an initial indication of phage lytic capacity (27, 28); for instance, virulent phages typically showed large, clear plaques with a short formation time, while temperate phages often had small, turbid plaques with a prolonged formation time. Undoubtedly, systematic exploration of *Vibrio*-associated temperate phage populations or individuals would be of great significance to improve our understanding of bacterial pathogenesis, evolution, and ecology.

### HGT drives phage diversification and adaptation of host recognition mechanisms

Phage morphotypes were predicted using Virfam (29) and verified through comprehensive genome analysis, which identified nine myophages (with long, contractile tails), two siphophages (with long, non-contractile tails), and two podophages (with short, non-contractile tails). Notably, taxonomic classification revealed that two siphophages (phiCAIM148 and phiFDAARGOS107) and one myophage (phiCAIM463) belong to the same Mu-like subfamily but different genera (Fig. 5a). Comparative genomic analysis suggested that this genus-level differentiation resulted from tail module recombination (Fig. 4), leading to altered adsorption apparatuses, particularly receptor-binding proteins (typically tail fibers or spikes) that were crucial for host recognition (30).

For Mu-like siphophages (represented by phiFDAARGOS107), structural modeling showed that GP41 and GP42 were similar to tail fiber-forming proteins GP47 and GP48 of *Pseudomonas* phage JBD30, with root mean square deviation (RMSD) values of 3.12 and 5.26, respectively (Fig. S1a). Cryo-electron microscopy analysis of JBD30 revealed three tail fibers, each comprising three GP47-GP48 heterodimers (#PDB: 8RK5; Fig. S1b), forming pilus interaction interfaces (31). This suggests that these Mu-like siphophages might similarly employ type IV pili for initial host cell recognition (31). The Mu-like myophage phiCAIM463 encoded a three-domain tail spike protein (TSP, GP52): an N-terminal “stem” domain for tail attachment, a central “neck” domain, and a C-terminal “head” domain featuring an extended right-handed β-helix with depolymerase activity for capsular polysaccharide degradation (32) (Fig. S2a). While the stem domain of phiCAIM463 TSP shared sequence/structure similarity with its equivalent in *Vibrio splendidus* myophage martha 12B12 (a phiCAIM463 subfamily relative), the other regions showed no conservation. Notably, the head domain of martha 12B12 TSP contained two receptor-binding sites predicted to target ester-modified lipids or polysaccharides on the host cell membrane (33) (Fig. S2b). These findings collectively highlighted how HGT drove phage diversification and adaptation of host recognition mechanisms.

### Putative virulence factors encoded by *V. harveyi* prophages

To search for potential prophage-encoded VFs, we compared the amino acid profiles of these prophages against two established databases: the Virulence Factor Database (VFDB core set A) (34) that contained experimentally validated VF-associated genes and the Comprehensive Antibiotic Resistance Database (CARD) (35). Though no antibiotic-resistant genes were recognized, this analysis detected several prophage-encoded VFs (Table 1).

**TABLE 1:** Prophage-encoded homologs of experimentally validated virulence determinants

Prophage-encoded VFs	Representative VFs	Species	Product	VFDB category^*a*^	BLASTp		
					%Identity	E-value	Score
phi345 gb|WP_101904743.1	VFG043629 gb|WP_01108107.1	*Vibrio vulnificus* CMCP6	Flp family type IVb pilin	Flp pili (VF0612)—adherence (VFC0001)	40.8	1.18E−07	42.4
phiGANI1709 gb|WP_110415004.1					40.8	6.30E−07	40.4
pAOD131/pGANI1807 gb|WP_017817514.1	VFG000077 gb|NP_465991	*Listeria monocytogenes* EGD-e	ATP-dependent Clp protease proteolytic subunit	ClpP (VF0074)—stress survival (VFC0282)	33.1	9.95E−15	70.9
phi345 gb|WP_101904720.1	Xf-VapD gb|AE003851_51	*Xylella fastidiosa* 9a5c	Thermostable ribonuclease	N/A	39.8	7E−20	65.5
phiCAIM148/phiFDAARGOS107 gb|WP_050940436.1	VpaChn25_0724 gb|WP_065870376.1	*Vibrio parahaemolyticus* CHN25	Phenolic acid decarboxylase regulator, PadR	N/A	74.5	7E−55	152
phiCAIM463 gb|WP_050926363.1					75.3	2E−58	161
pCAIM1508 gb|WP_114091696.1					N/A	N/A	N/A
phi345/phiGANI1709 gb|AWB00219.1					N/A	N/A	N/A

^*a*^ The virulence factor category is determined based on the content described in the “VFs description file” exported from VFDB (http://www.mgc.ac.cn/VFs/; last updated on Friday, 14 March 2025, 19:30:01). N/A, not included in the VFDB.

The Flp (fimbrial low-molecular-weight protein) family of type IV pili, encoded by phi354 and phiGAN1709, was identified as an adhesion-related VF (accession number: VF0612). As a key component of the “tight adherence (Tad) pilus” (type IVc), Flp pilin was typically involved in bacterial adherence and biofilm formation. In *Vibrio vulnificus*, Flp pilin significantly contributed to virulence by facilitating host cell and tissue invasion, bloodstream survival, and resistance to serum bactericidal activities (36). In addition, ATP-dependent Clp protease proteolytic subunit (ClpP), encoded by pAOD131 and pGAN1807, was identified as a VF for stress survival (VF0074). ClpP influenced bacterial virulence through multiple mechanisms, including stress adaptation, biofilm formation, and virulence factor expression, making it a promising antimicrobial target (37).

Additional prophage-encoded VFs with homology to known virulence determinants but were not documented in the VFDB included VapD (virulence-associated protein D), uniquely present in phi354, and PadR (phenolic acid decarboxylase regulator), widely distributed among *V. harveyi* prophages. VapD functioned as a thermostable ribonuclease, with elevated expression linked to biofilm development in the virulent *Xylella fastidiosa* strain (38). PadR, which regulated genes involved in detoxification, virulence, and multidrug resistance (39), was remarkably conserved across all *V. harveyi* Mu-like prophages and the prototype phage Mu (GP26). A homolog (VpaChn25_0724) from a Mu-like prophage within *V. parahaemolyticus* strain CHN25 had been experimentally shown to affect host cell motility, biofilm formation, and cytotoxicity (40). Comparisons of predicted structures between Mu GP26, VpaChn25_0724, and the Mu-type PadR identified in this study revealed remarkable similarity (Fig. S3). Interestingly, we also identified PadR-like homologs in non-Mu-like prophages (phi345, phiGANI1709, and pCAIM1508) that, while lacking sequence similarity to Mu-type PadR, showed weak structural resemblance. With six representatives spanning three subfamilies and four genera, PadR emerged as the most prevalent VF among *V. harveyi* prophages. To sum up, these findings indicate the significance of PadR-like factors in bacterial virulence and ecological adaptation.

### Prophages with varying prevalence: widely distributed, clade-specific, and strain-specific

To provide a comparative framework for the analyzed *V. harveyi* prophages, we compiled prophage genomes from 688 high-quality *Vibrio* spp. genomes (hereinafter referred to as “vibrioprophages”). Given the challenges of manual inspection at this scale, we implemented an automated tool for prophage identification. By comparing the intersection-over-union ratio (i.e., the ratio of the intersection length of the predicted region and the actual prophage region to the union length of both), geNomad performed the best in prediction completeness and accuracy (geNomad: 92.3% ± 13.3%, Virsorter2: 40.8% ± 9.3%, and CheckV: 80.2% ± 17.2%) (Fig. 2), and thus was selected for systematic prophage screening. The resulting 515 predicted vibrioprophages and phage genomes from ProkaryoticViralRefSeq211 were analyzed for proteome-based vConTACT2 similarity (41) in comparison with the *V. harveyi* prophages. Among reference phage sequences forming predicted viral clusters (equivalent to genus-level groupings [41]) with the analyzed *V. harveyi* prophages, 101 members were predicted vibrioprophages, while only 4 were temperate vibriophages from the RefSeq database (Data set S1). Such numerical disparity (101:4) highlighted the current paucity of characterized temperate vibriophages in public databases.

**Fig 6:**
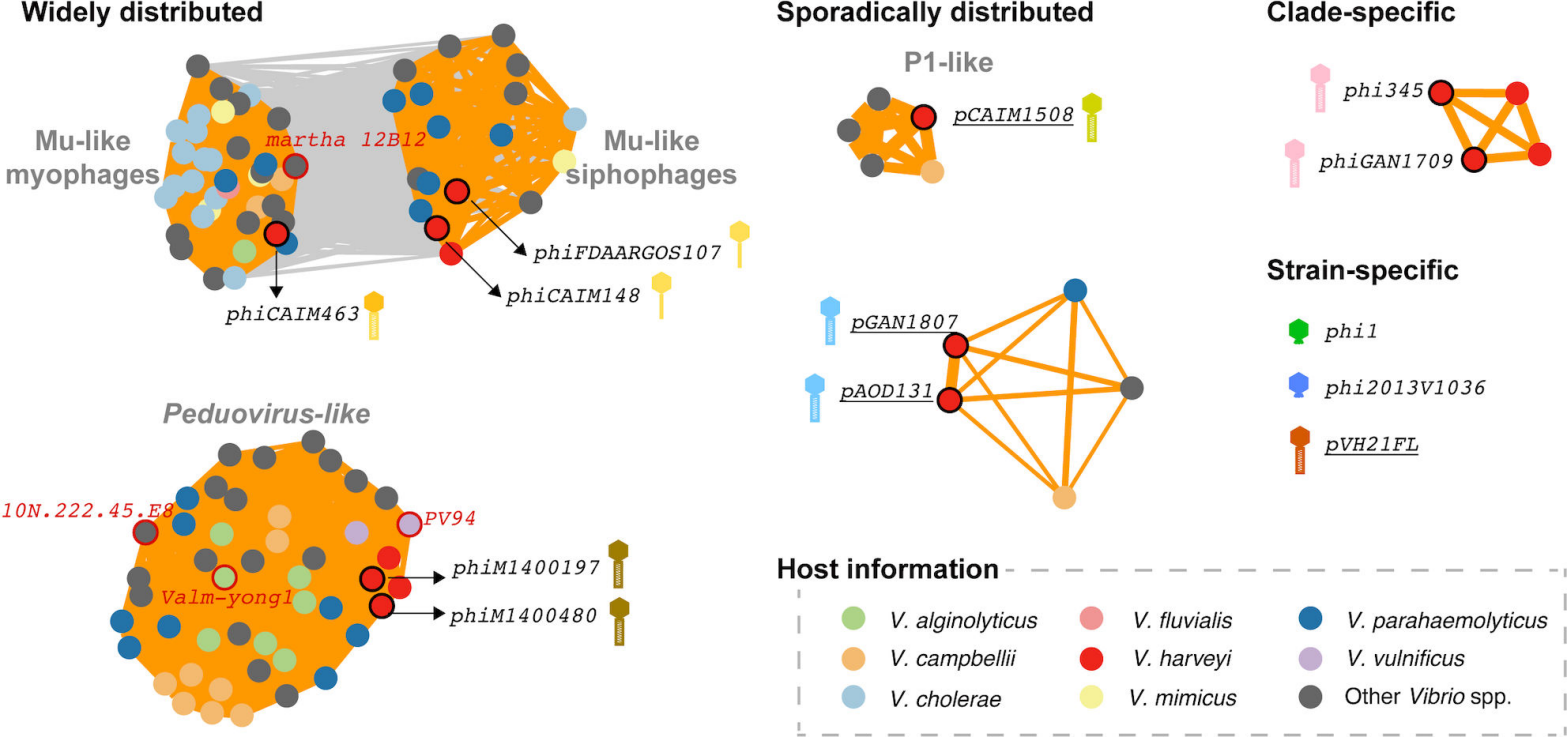
Protein-sharing network showing hierarchical prevalence patterns among *V. harveyi* prophages. Each node represented a phage genome and was color-coded corresponding to host taxonomic classification, with border styles indicating phage origin: black borders, *V. harveyi* prophages analyzed in this study; red borders, reference phage genomes from ProkaryoticViralRefSeq211; no borders, predicted vibrioprophages from 688 *Vibrio* spp. genomes. Edge thickness scales with vConTACT2 similarity scores, with edges connecting phages within the same viral cluster highlighted in orange.

Network analysis revealed hierarchical prevalence patterns among prophage genera (Fig. 6). Mu-like myophages/siphophages and *Peduovirus*-like prophages showed broad occurrence across multiple *Vibrio* species. Plasmid-phages pCAIM1508, pGAN1807, and pAOD131 had several genus-level homologs identified from other *Vibrio* species, suggesting their sporadical distribution pattern. In contrast, phi345 and phiGAN1709 clustered exclusively with two other *V. harveyi* prophages, likely being clade-specific for *V. harveyi*. Prophages phi1, phi2013V1036, and pVH21FL appeared as singletons without forming high-confidence viral clusters with any (pro)phages, implying possible strain-specific adaptations. These findings suggested that, in addition to existing phage isolation bias, the observed diversity gap in *V. harveyi* prophages may reflect their host-specific evolution.

The prevalence patterns of *V. harveyi* prophages suggested their different roles in host virulence or ecological adaptation. The universal presence of PadR-like homologs in all Mu-like siphovibriophages (*n* = 18) and myovibriophages (*n* = 37) implied that this putative virulence factor might influence *Vibrio* population dynamics at large. Virulence-related factors encoded by clade-specific prophages, such as Flp pili in phi345-like prophages, might contribute to *V. harveyi*-specific adaptations. Future studies investigating whether prophage prevalence patterns correlate with host-specific or clade-specific phenotypes or ecological niches would be of importance.

### *In-silico* assessment of prophage lytic potential

Prophages possessed the capacity for lysogeny-lysis switching and might be spontaneously released or induced by external stimuli (11, 42). To identify historically active prophages, we compared their genomes against CRISPR (clustered regularly interspaced short palindromic repeats) spacers predicted from 55 *V*. *harveyi* and 688 *Vibrio* spp. genomes. The CRISPR-Cas systems, present in most bacteria and archaea, provided adaptive immunity through *cas* genes and spacer arrays (43)—these spacers, derived from prior viral or plasmid invaders, conferred resistance against subsequent infections by matching viruses or plasmids.

**Fig 7:**
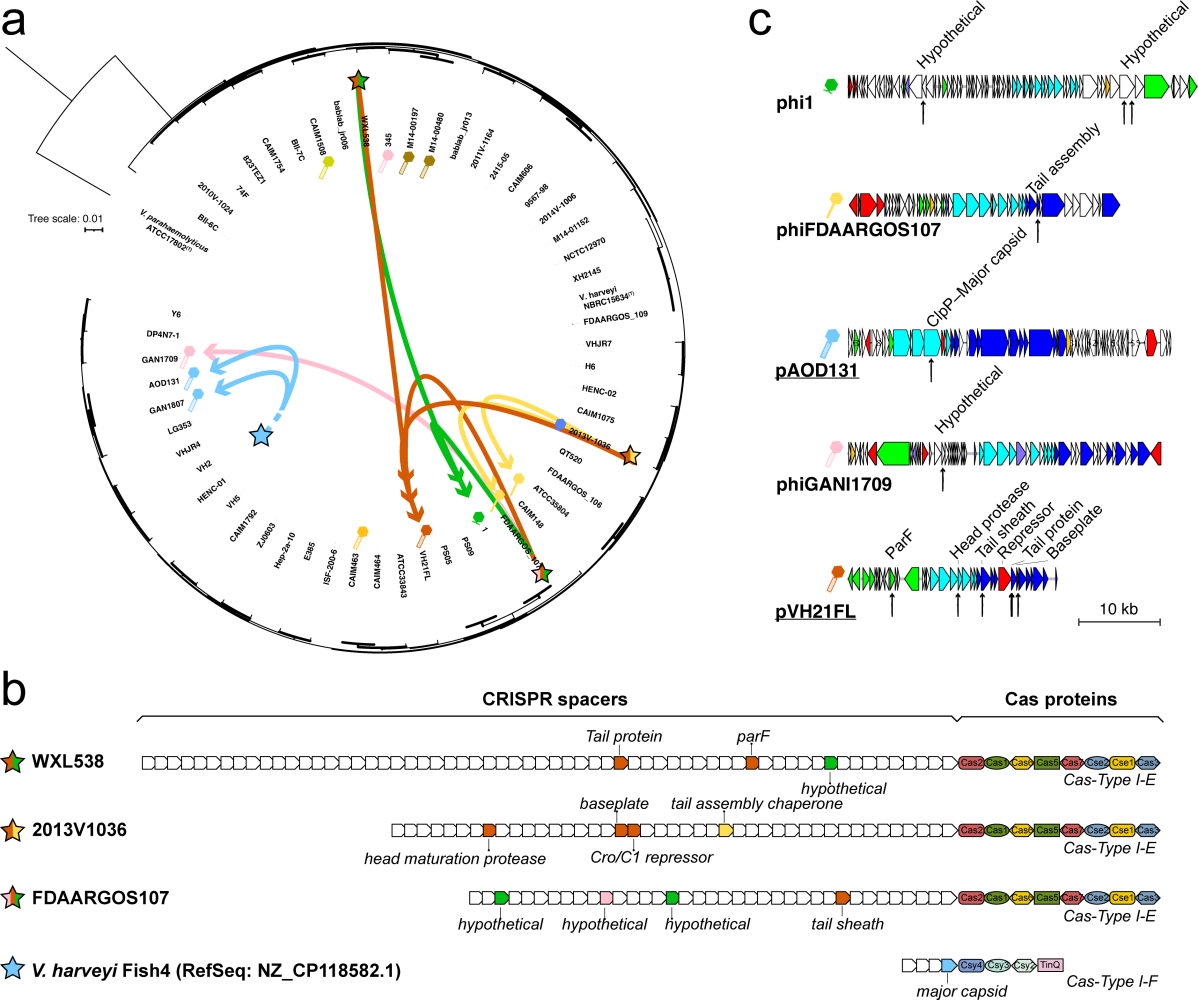
Evidence of historical phage infections through CRISPR spacer matching. (**a**) CRISPR-Cas systems (star symbols) targeting *V. harveyi* prophages, including one CRISPR-Cas system (blue star) from an external *V. harveyi* genome (NZ_CP118582.1). (**b**) Structural organization of identified Type I-E/I-F CRISPR arrays, with spacers color-coded by the genus of the prophage target. Each targeting prophage gene was annotated accordingly. (**c**) Phage genome maps (formatted as in Fig. 4) highlighting spacer target sites (black arrowheads).

Our analysis revealed that over half of *V. harveyi* prophages (7/13) were targeted by 12 non-redundant spacers originating from three Cas-Type I-E systems (identified in 3/55 *V*. *harveyi* genomes) and 1 Cas-Type I-F system (from a recently publicly available *V. harveyi* genome) (Fig. 7a and b; Data set S2). The plasmid-phage pVH21FL was targeted most frequently, matching six spacers across all three Cas-Type I-E systems (Fig. 7c). Our analysis highlighted historical lytic activity among *V. harveyi* prophages and their potential for horizontal transfer of VFs to susceptible hosts or co-infecting (pro)phages. While two Mu-like siphophages (phiFDAARGOS107 and phiCAIM148) showed spacer targeting in their tail assembly genes, the Mu-like myophage (phiCAIM463) remained untargeted. This suggests that, in addition to the diversification of host recognition mechanisms, HGT-mediated tail replacement could represent an evolutionary strategy to evade CRISPR spacer recognition. To examine potential infection events occurring in more distantly related hosts, we compared prophage genomes against spacers entries in CRISPRCasdb (version 14 April 2022), a database containing CRISPR spacer data from >16,990 complete prokaryotic genomes (44). However, no additional matches were detected, confirming that the identified infection histories were specific to *V. harveyi,* while no evidence existed for current spacer matches from non-*V*. *harveyi* hosts.

### Conclusions

Leveraging prophage border curation and bioinformatic techniques, this study established a genomic data set of tailed prophages targeting *Vibrio harveyi*. Our analysis revealed diverse parasitic strategies and hierarchical distribution patterns among these prophages, illustrating their adaptive plasticity in host exploitation. The finding that half of prophage subfamilies constituted novel taxonomic units underscored significant underrepresentation of temperate vibriophages in existing databases—a diversity gap jointly driven by host-specific temperate phage evolution and isolation biases favoring virulent phages for therapeutic purposes. Importantly, the widespread distribution of PadR-like transcriptional regulators and other virulence homologs suggested that prophages served as reservoirs for adaptive traits driving *Vibrio* pathogenesis. Future bridging of genomic predictions with functional characterization—particularly for clade-specific prophages and their virulence determinants—will be essential for developing phage-based interventions against luminous vibriosis.

## MATERIALS AND METHODS

### Analysis of *Vibrio harveyi* genomes

Fifty-nine genomes labeled “*V. harveyi*” were retrieved from the NCBI RefSeq database (accessed 1 March 2023). Initial species verification was performed using genome-based DDH analysis via Genome-to-Genome Distance Calculator (GGDC 3.0) (14), with 55 genomes confirming *V. harveyi* identity (DDH similarity > 70%) and retained for subsequent analyses (Table S1). A phylogenetic tree was constructed using concatenated sequences of 92 single-copy core bacterial genes extracted by UBCG 3.0 (45), with visualization and annotation performed in iTOL 6 (46).

### Prophage identification and border curation

Prophages were identified through combined manual inspection and computational prediction. Bacterial open reading frames (ORFs) were initially predicted via the online RAST server (47). Clustered phage-related genes from annotation files were flagged as prophage candidates. We supplemented the analysis with three widely used phage detection tools, CheckV, Virsorter2, and geNomad (16–18). Precise boundaries of each candidate were determined via BLASTn comparison between parental bacterial genome and an isogenic prophage-free reference genome, such that host sequences flanking the prophage were precisely removed. Complete genomic coordinates for all identified prophages are provided in Table S2.

### Phage bioinformatic analysis

Prophage ORF annotation employed BLASTp, InterProScan (48), and HHpred (49). Putative virulence factors and antibiotic-resistant genes were identified by querying VFDB (34) and CARD (35), respectively, using BLASTp with thresholds of E-value < 10^−5^ and score > 40. Phage gene maps were generated using custom JavaScript. Protein structures were predicted using AlphaFold 3 (50), visualized in PyMOL (51), and aligned with the RMSD value calculated. Conserved residues in aligned protein objects were highlighted by cartoon putty radius, using the “color_by_conservation” script (https://pymolwiki.org/index.php/Color_by_conservation).

### Phage morphotype prediction

Phage morphotypes were predicted using Virfam (29) and verified through genomic examination. Myophages and siphophages possessed complete tail machinery components, including major tail proteins, tail tape measure proteins, and tail fibers. Myophages were further characterized by the presence of tail sheath proteins that enable contractile motion (29). In contrast, podophages were characterized by their minimal tail-related gene content, with tail fibers being the principal recognizable components. Phage receptor-binding proteins (mainly tail fibers or spikes) were predicted using PhageRBPdetect v4 (52) and subsequently verified through manual inspection.

### Phage taxonomic classification

Proteome-based phylogeny was constructed using the VipTree server (24), with the phage genomes from the RefSeq database (release 220) as references. Taxonomic classification employed established thresholds: VipTree branch lengths of 0.05 and 0.1 for family and subfamily delineation (26), respectively, and average nucleotide identities of 95% and 70% for species and genus classification (25), respectively. Pairwise average amino acid identity was calculated using CompareM (https://github.com/dparks1134/CompareM), with a minimum of 10% orthologous genes as an empirical criterion for delineating phage families. The protein-sharing heatmap was visualized using the R package pheatmap.

### Network analysis

To create comparative prophage profiles, 688 *Vibrio* spp. genomes with complete or chromosome-level assemblies were retrieved from the NCBI RefSeq database (accessed 9 September 2023), and prophage prediction was performed using geNomad (18) with optimized parameters for *Caudoviricetes* phages (“viral genes” ≥ 30, “virus score” ≥ 0.8). The resulting 515 virbioprophages, the analyzed *V. harveyi* prophages, and reference phage genomes from RefSeq (version 211), were analyzed using vConTACT2 (41) to establish proteomic similarity networks (Data set S1). Network visualization in Cytoscape 3.8.0 (53) employed a prefuse force-directed layout weighted by vConTACT2 similarity scores, with node proximity reflecting shared protein cluster content.

### CRISPR spacer matching

To probe into historical phage infection events, we investigated whether prophage fragments have been preserved as immunological records in CRISPR-Cas systems. Spacer sequences were identified from 55 *V*. *harveyi* and 688 *Vibrio* spp. genomes using MinCED (54), and only CRISPR-Cas systems with evidence levels 3 or 4, as assessed by CRISPRCasFinder (55), were considered. To examine potential infection events occurring in more distantly related hosts, prophage genomes were queried against spacers entries in CRISPRCasdb (6 April 2022 update) that documents spacer data from >16,990 prokaryotic genomes (44). CRISPR spacer matching was conducted with stringent criteria requiring 100% identity and 100 coverage (BLASTn) for positive identification (Data set S2).

## Data Availability

The data sets supporting the conclusions of this article are included within the article and its additional files.

## SUPPLEMENTAL MATERIAL

Data set S1 msphere.00228-25-s0001.xlsx vConTACT2-predicted viral clusters and pairwise similarities scores for phage genomes.

Data set S2 msphere.00228-25-s0002.xlsx Comprehensive list of CRISPR spacer-matched genes across *V. harveyi* prophages.

Fig. S1 msphere.00228-25-s0003.pdf Structural characterization of Mu-like siphophage phiFDAARGOS10 tail fibers.

Fig. S2 msphere.00228-25-s0004.pdf Structural analysis of TSPs from Mu-like myophages.

Fig. S3 msphere.00228-25-s0005.pdf Structural comparison of PadR-like transcriptional regulators.

Table S1 msphere.00228-25-s0006.pdf Metadata for the analyzed *V. harveyi* genomes.

Table S2 msphere.00228-25-s0007.pdf Genomic coordinates of identified *V. harveyi* prophages.

ASM does not own the copyrights to Supplemental Material that may be linked to, or accessed through, an article. The authors have granted ASM a non-exclusive, world-wide license to publish the Supplemental Material files. Please contact the corresponding author directly for reuse.
