# Supplementary material for: Prevalence, diversity, and parasitism of tailed prophages in Vibrio harveyi
Source: mSphere. 2025 Aug 25;10(9):e00228-25. doi: 10.1128/msphere.00228-25 (PMC12482185; doi:10.1128/msphere.00228-25)
Supplement: Fig. S1 — Structural characterization of Mu-like siphophage phiFDAARGOS10 tail fibers. [file msphere.00228-25-s0003.pdf]

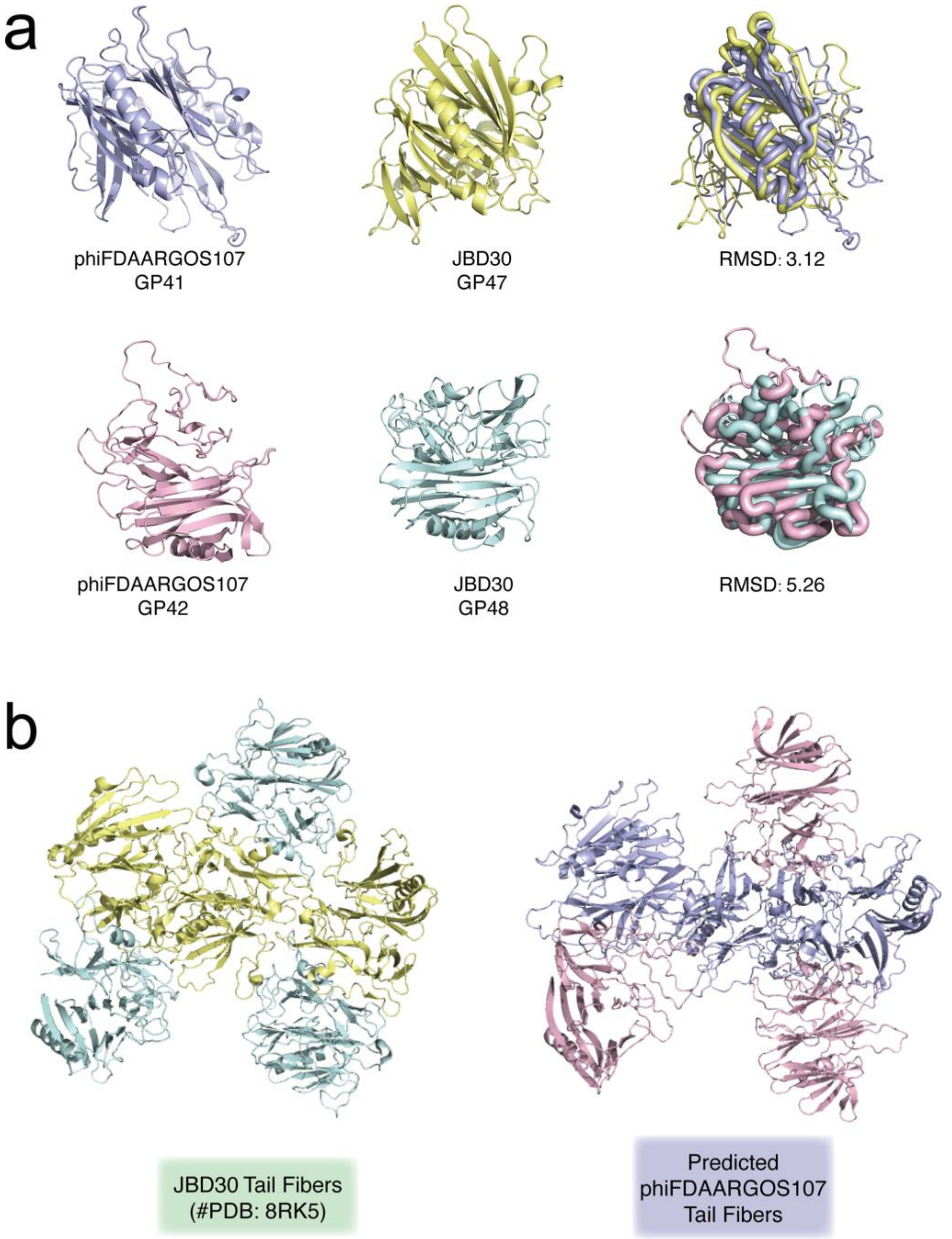

Fig. S1 Structural characterization of Mu-like siphophage phiFDAARGOS10 tail fibers. (a) Structural alignment of predicted tail fiber-forming proteins GP41 and GP42 from phiFDAARGOS10 with their homologs GP47 and GP48 from *Pseudomonas* phage JBD30, with root-mean-square deviation (RMSD) values indicating structural similarity. Conserved residues in aligned protein objects were highlighted by cartoon putty radius to enhance visualization effect. (b) Comparative architecture of tail fiber complexes: the left panel showed the crystallographically resolved structure of JBD30 tail fiber (#PDB: 8RK5) composed of three GP47–GP48 heterodimers; the right panel presented the predicted tail fiber organization of phiFDAARGOS10.
