# Supplementary material for: Prevalence, diversity, and parasitism of tailed prophages in Vibrio harveyi
Source: mSphere. 2025 Aug 25;10(9):e00228-25. doi: 10.1128/msphere.00228-25 (PMC12482185; doi:10.1128/msphere.00228-25)
Supplement: Fig. S2 — Structural analysis of TSPs from Mu-like myophages. [file msphere.00228-25-s0004.pdf]

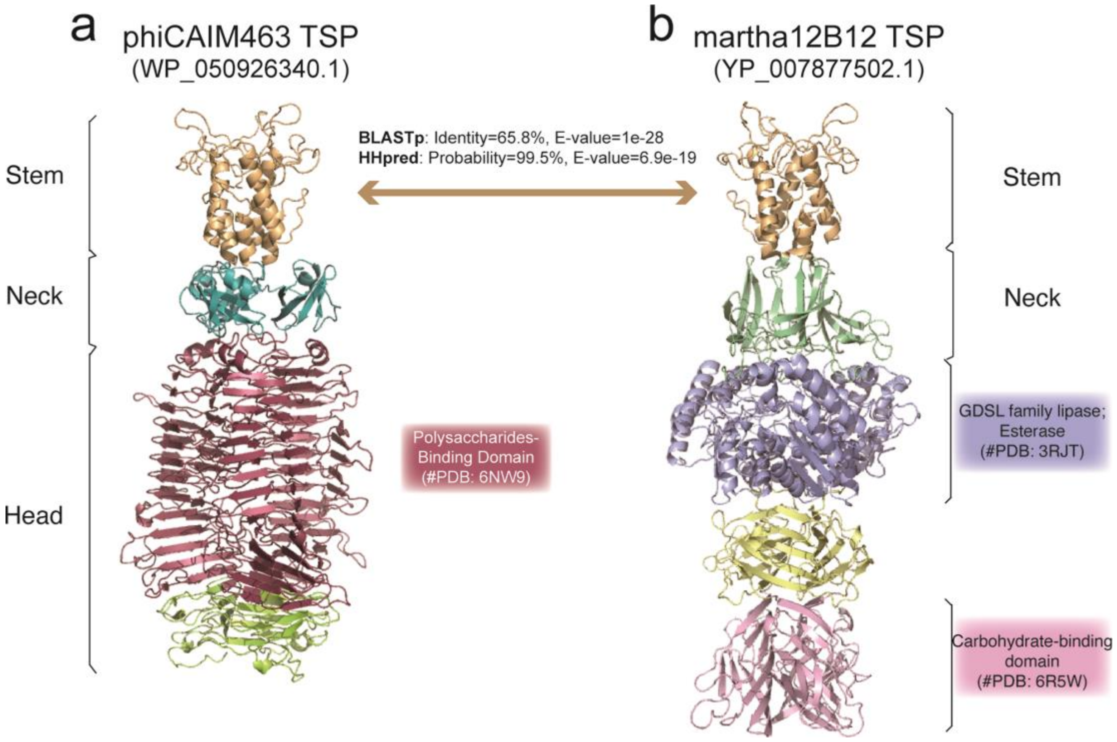

Fig. S2 Structural analysis of tail spike proteins (TSPs) from Mu-like myophages (a, *V. harvei*-infecting phiCAIM463) and (b, *V. splendidus*-infecting martha 12B12). Predicted homotrimeric TSP structures were shown as domain-colored ribbons with annotated receptor-binding sites.
