# Supplementary material for: Prevalence, diversity, and parasitism of tailed prophages in Vibrio harveyi
Source: mSphere. 2025 Aug 25;10(9):e00228-25. doi: 10.1128/msphere.00228-25 (PMC12482185; doi:10.1128/msphere.00228-25)
Supplement: Fig. S3 — Structural comparison of PadR-like transcriptional regulators. [file msphere.00228-25-s0005.pdf]

Mu-type PadR-like proteins

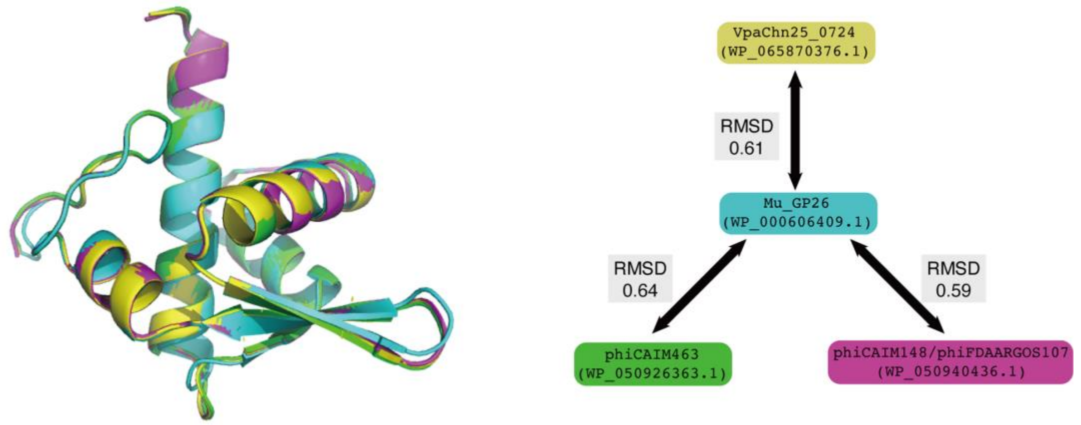

Non-Mu-type PadR-like proteins

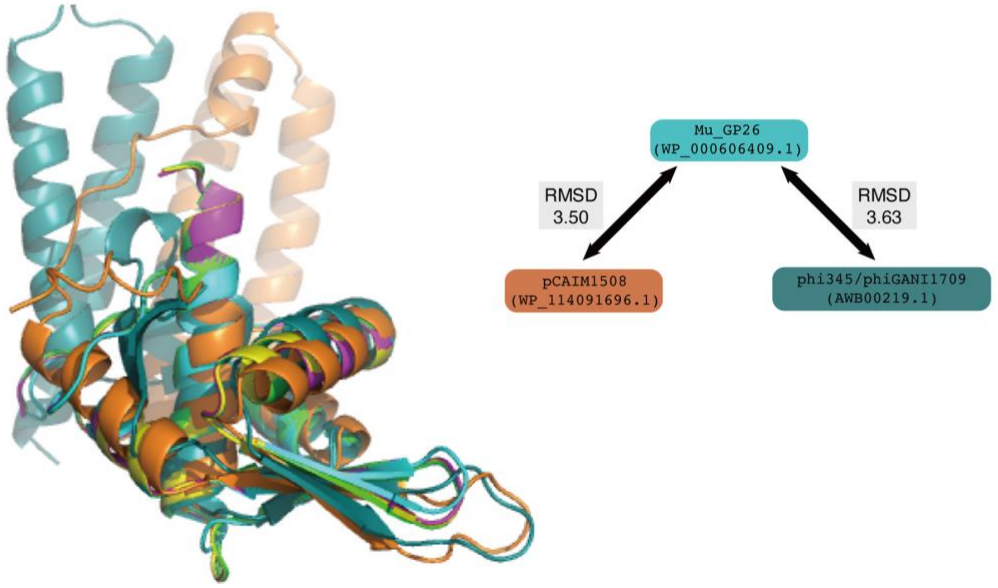

Fig. S3 Structural comparison of PadR-like transcriptional regulators. The upper panel displayed the superimposed structures of Mu-type PadR homologs, with pairwise RMSD values provided. The lower panel displayed superimposed structures of Mu-type and non-Mu-type PadR homologs.
