# Supplementary material for: Prevalence, diversity, and parasitism of tailed prophages in Vibrio harveyi
Source: mSphere. 2025 Aug 25;10(9):e00228-25. doi: 10.1128/msphere.00228-25 (PMC12482185; doi:10.1128/msphere.00228-25)
Supplement: Table S1 — Metadata for the analyzed V. harveyi genomes. [file msphere.00228-25-s0006.pdf]

Table S1. Metadata for the analyzed 55 *V. harveyi* genomes.

Strain	Assembly Accession	Sampling Year	Geographic Location	Host
1	GCF_024971935.1	2018/6/10	China: Guangdong	*Penaeus vannamei*
345	GCF_002850295.1	2013/10	China: Shenzhen	diseased Epinephelus oanceolutus
2415-05	GCF_001185545.1	2005	USA: Hawaii	Homo sapiens
9567-98	GCF_014525075.1	1998	N/A	N/A
2010V-1024	GCF_012275085.1	2010/5/12	USA: Florida	N/A
2011V-1164	GCF_009665315.1	2011	N/A	N/A
2013V-1036	GCF_014525195.1	2013	N/A	N/A
2014V-1006	GCF_014525215.1	2014	N/A	N/A
74F	NZ_JPTL01000001.1–NZ_JPTL01002036.1	2007	Brazil: Abrolhos Bank	diseased coral
823TEZ1	GCF_000818375.1	2005	Japan: Taisei	moribund *Haliotis discushannai* (abalone)
AOD131	GCF_000347555.1	N/A	Taiwan: Kaohsiung	*Epinephelus lanceolatus*
ATCC33843	GCF_000770115.1	1971	N/A	N/A
ATCC35804	GCF_001185495.1	1982	USA: Baltimore, Maryland	*Carcharhinus plumbeus*
bablab_jr006	GCF_016464165.1	2017/11	Cuba: Gardens of the Queen	*Diploria labyrinthiformi*
bablab_jr013	GCF_016464085.1	2017/11	Cuba: Gardens of the Queen	*Orbicella faveolata*
BII-6C	GCF_024531115.1	2017/8/14	Mauritius: Pointe aux Piments	*Lethrinus nebulosus*
BII-7C	GCF_024530995.1	2017/8/14	Mauritius: Grand Gaube	*Epinephelus fasciatus*
CAIM1075	GCF_001185575.1	2003	Mexico: SCPA Burabampo SCL	*Crassostrea gigas* (oyster)
CAIM148	GCF_001185585.1	1995	Mexico: Valle de Matatipac	diseased shrimp

Table S1–*continued*

Strain	Assembly Accession	Sampling Year	Geographic Location	Host
CAIM1508	GCF_003326605.2	2005/5/9	Mexico: Mazatlan	*Sphoeroides annulatus*
CAIM1754	GCF_003334845.1	2005/5/9	Mexico: Mazatlan	*Sphoeroides annulatus*
CAIM1792	GCF_000259935.1	2005/7/18	Mexico	diseased *Penaeus vannamei*
CAIM463	GCF_001185405.1	1991	Greece	*Dicentrarchus labrax* (sea bass)
CAIM464	GCF_001185645.1	1990	Spain	*Scophthalmus maximus* (turbot)
CAIM606	GCF_001185455.1	1992	Japan: Uchiura Bay, Numazu	*Trachurus japonicus* (Japanese horse mackerel)
DP4N7-1	GCF_019800715.1	2020/8/24	China: Shenzhen	sediment
E385	GCF_000493315.1	2009	China: Daya Bay of Guangdong Province	diseased *Epinephelus coioides*
FDAARGOS_106	GCF_001525585.2	1982	USA: Maryland	Shark
FDAARGOS_107	GCF_001558435.2	N/A	Bahamas	Shark
FDAARGOS_109	GCF_001471575.2	1935	USA: Maryland	dead amphipod
GAN1709	GCF_003203495.1	2017/9/6	Japan: Kochi	*Seriola dumerili*
GAN1807	GCF_006538425.1	2018/7/11	Japan: Kochi	*Seriola dumerili*
H6	GCF_001185465.1	1992	Bahamas: Chub Cay	*Holocentrus* sp. (squirrelfish)
HENC-01	GCF_000305715.2	2010	Haiti	*Homo sapiens*
HENC-02	NZ_JH974191.1–NZ_JH975907.1	2010	Haiti	*Homo sapiens*

Table S1–*continued*

Strain	Assembly Accession	Sampling Year	Geographic Location	Host
Hep-2a-10	GCF_001718025.1	2014/09	USA: Flour Bluff, Texas	*Penaeus vannamei*
ISF-200-6	GCF_024745675.1	2015	Viet Nam	raw shrimp
LG353	GCF_002105235.1	2016/6	China: Hainan	*Trachinotus ovatus*
M14-00197	GCF_022397835.1	2014/1/1	Australia: NSW, Port Stephens	*Magallana gigas*
M14-00480	GCF_022397815.1	2014/1/1	Australia: NSW, Port Stephens	*Magallana gigas*
M14-01152	GCF_022397925.1	2014/1/1	Australia: NSW, Port Stephens	*Magallana gigas*
NBRC15634	GCF_000400305.1	1935	USA: Maryland	dead amphipod
NCTC12970	GCF_900460165.1	1935	USA: Maryland	dead amphipod
PS05	GCF_022397875.1	2014/1/1	Australia: NSW, Port Stephens	*Magallana gigas*
PS09	GCF_022395475.1	2014/1/1	Australia: NSW, Port Stephens	*Magallana gigas*
QT520	GCF_001908435.2	2016/6/2	China: Chenmai qiaotou	diseased *Trachinotus ovatus*
VH2	GCF_001262735.2	2007	Greece: Crete	amberjack juveniles
VH5	GCF_001262745.1	2007	Greece: Crete	amberjack juveniles
VH21FL	GCF_028551315.1	2005	South Korea: Pohang	diseased *Paralichthys olivaceus*
VHJR4	GCF_001050875.1	2009	Malaysia	*Lates calcarifer*
VHJR7	GCF_000442925.1	2009	Malaysia	*Lates calcarifer*
WXL538	GCF_009184745.1	2015/10	China: East China Sea	seawater at 25-m depth
XH2145	GCF_021397735.1	2021	China: Hangzhou	seawater
Y6	GCF_002864745.1	2015	Viet Nam	*Lates calcarifer*
ZJ0603	GCF_000275705.1	2012	China: Guangdong	diseased *Epinephelus coioides*
