# Supplementary material for: Prevalence, diversity, and parasitism of tailed prophages in Vibrio harveyi
Source: mSphere. 2025 Aug 25;10(9):e00228-25. doi: 10.1128/msphere.00228-25 (PMC12482185; doi:10.1128/msphere.00228-25)
Supplement: Table S2 — Genomic coordinates of identified V. harveyi prophages. [file msphere.00228-25-s0007.pdf]

Table S2. Genomic coordinates of identified *V. harveyi* prophages.

Prophage[a]	Parasitism	Genome Length (bp)	Prophage Coordinates (bp)		
			Contig Accession Number	Start	End
phi1	Integrated	43,581	NZ_CP080098.1	3,532,037	3,575,617
phi2013V1036	Integrated	37,238	NZ_JACGMA010000003.1	1,841,400	1,878,637
phi345	Integrated	39,620	NZ_CP025537.1	2,714,250	2,753,869
phiGAN1709	Integrated	38,737	NZ_BGNF01000011.1	34,371	73,107
phiM1400197	Integrated	31,548	NZ_JAKEUN010000003.1	17,746	49,293
phiM1400480	Integrated	31,548	NZ_JAKEUM010000004.1	17,746	49,293
phiCAIM463	Integrated	33,290	NZ_JPTP01000148.1	32,544	65,833
phiFDAARGOS107	Integrated	33,645	NZ_CP014039.2	2,253,119	2,286,763
phiCAIM148_contig1	Integrated	218	NZ_JPTN01000185.1	1	218
phiCAIM148_contig2		19,514	NZ_JPTN01000611.1	1	19,514
phiCAIM148_contig3		5,939	NZ_JPTN01000402.1	1	5,939
phiCAIM148_contig4		268	NZ_JPTN01000621.1	1	268
phiCAIM148_contig5		3,853	NZ_JPTN01000853.1	1	3,853
phiCAIM148_contig6		1,380	NZ_JPTN01000260.1	1	1,380
phiCAIM148_contig7		802	NZ_JPTN01000781.1	4,391	5,192
pAOD131	Plasmid-like	39,771	NZ_AOMR01000041.1	1	39,771
pGAN1807	Plasmid-like	39,064	NZ_BJKR01000024.1	1	39,064
pCAIM1508	Plasmid-like	96,783	NZ_QOUW02000007.1	1	96,783
pVH21FL	Plasmid-like	25,664	NZ_CP117057.1	1	25,664

[a]Prophage nomenclature follows: "phi" for integrated prophages and "p" for plasmid-phages, followed by host strain identifiers.
